# Antimicrobial Resistance Surveillance in Post-Soviet Countries: A Systematic Review

**DOI:** 10.3390/antibiotics13121129

**Published:** 2024-11-25

**Authors:** Dariga Zhazykhbayeva, Dinagul Bayesheva, Zhanar Kosherova, Yuliya Semenova

**Affiliations:** 1 School of Medicine, Nazarbayev University, Astana 010000, Kazakhstan; dariga.zhazykhbayeva@nu.edu.kz (D.Z.); yuliya.semenova@nu.edu.kz (Y.S.); 2 Department of Paediatric Infectious Diseases, NJSC “Astana Medical University”, Astana 010000, Kazakhstan; baesheva.d@amu.kz

**Keywords:** antimicrobial resistance surveillance, national action plan, post-Soviet countries

## Abstract

Background: Antimicrobial resistance (AMR) is a global health threat. AMR surveillance is crucial for understanding and controlling the spread of AMR. Surveillance systems can inform clinicians, guide health policymakers, and support effective AMR interventions. AMR surveillance data from former Soviet region countries are often limited, resulting in gaps in up-to-date knowledge. Methods: This systematic review was registered under the PROSPERO protocol CRD42024537799 and followed the PRISMA guidelines. Data from five databases (PubMed, MEDLINE, Embase, CINAHL, and CyberLeninka) and official sources were searched according to the inclusion criteria. Results: In total, 30 publications describing AMR surveillance and National Action Plans (NAPs) were included. Among the 15 countries, 14 (93.3%) have either developed a NAP or are in the process of implementing one; and 7 (46.7%) countries have a standardized AMR surveillance system. Almost all countries have reference laboratory centers, but nine (60%) countries have established standard testing methods in all sites. Only three (20%) countries have fully implemented quality assessment. There is an increasing tendency to involve and report AMR data to international networks, and countries will strengthen their AMR systems by adhering to international standards. The subgroup analysis revealed that Central and Western Asian countries are less developed in terms of AMR surveillance, which may encourage proactive engagement in AMR governance in these regions. Conclusions: This review is crucial for understanding the current efforts and improving AMR surveillance in former Soviet countries. The findings are promising and indicate that AMR surveillance is established in all reviewed countries, although at different levels.

## 1. Introduction

Antimicrobial resistance (AMR) is an acquired feature of microorganisms such as bacteria, viruses, fungi, and parasites that results in a failure to respond to treatment. AMR is a global threat leading to increased mortality and morbidity rates across the globe and considerable expenditures for patients and hospitals [1,2]. The main cause of AMR transmission is the misuse and overuse of antimicrobial drugs, mainly antibiotics, in clinical settings and agriculture [3].

AMR surveillance is the process of data collection, analysis, and reporting about susceptibility and resistance to antimicrobial drugs. This information helps to guide treatment guidelines, antibiotic stewardship (AMS) interventions, infection prevention and control measures, and public health policies. To effectively reduce the AMR burden, the provided surveillance data should be of high quality, up-to-date, and comprehensive [4]. To achieve this goal, each country should have a sustainable national AMR surveillance system with the One Health approach, i.e., including data from humans, animals, and the environment [5]. The majority of countries have reported on established AMR surveillance systems. However, their effectiveness and integration with One Health differ [6].

As a call for action to combat AMR, the World Health Organization (WHO) launched the Global Action Plan on antimicrobial resistance (GAP) in 2015. There are five strategic goals described in GAP, including increasing awareness and understanding of AMR among healthcare workers and the general population, enhancing knowledge and collecting evidence via surveillance and research, decreasing infection incidence by establishing infection prevention and control systems, optimizing the use of antimicrobial medicines in humans and animals, and expanding economic cases for sustainable investment to solve this issue [7]. To communicate AMR data across countries and help countries develop national AMR surveillance systems, there are several international AMR surveillance networks focused on all countries or particular regions, such as GLASS (Global Antimicrobial Resistance Surveillance System), EARS-Net (European Surveillance of Antimicrobial Resistance), CAESAR (Central Asian and European Surveillance of Antimicrobial Resistance), and others [8]. According to the WHO GAP and CAESAR manual, to address AMR effectively, governance should prioritize the development of a national AMR action plan (NAP) with clearly stated goals. NAP development and implementation should be supported by the assigned AMR focal point and intersectoral coordinating mechanism, both with determined functions and robust communication channels. The surveillance system should include a national AMR surveillance group, a group of related policy makers, the scientific community, and professional societies. For the AMR surveillance network, a national AMR reference laboratory should be in place along with sentinel sites to report the national, multinational, and international AMR databases [7,9].

After the collapse of the Soviet Union (USSR) in 1991, fifteen independent countries were established, including Armenia, Azerbaijan, Belarus, Estonia, Georgia, Kazakhstan, Kyrgyzstan, Latvia, Lithuania, Moldova, Russia, Tajikistan, Turkmenistan, Ukraine, and Uzbekistan. Some of these countries maintain strong ties with the Russian Federation. In contrast, Estonia, Latvia, and Lithuania entered the European Union (EU) and North Atlantic Treaty Organization (NATO), and Georgia, Moldova, and Ukraine expressed interest in aligning with Western countries, following the Baltics’ footsteps [10]. Historically, the USSR maintained a comprehensive system of infectious disease surveillance and control, however, after its dissolution, established countries faced challenges in sustaining infection prevention and control systems [11].

Among the fifteen former Soviet countries, the following four are classified by the World Bank as high-income countries (HICs): Latvia, Estonia, Lithuania, and the Russian Federation. The remaining eight countries are classified as upper-middle-income countries (UPICs), and three are classified as lower-middle-income countries (LMICs) [12]. According to Naghavi et al., Eastern Europe has the highest impact of AMR on mortality across the WHO European region. Their study estimated approximately 74.0 associated deaths per 100,000 population. When the authors analyzed age-standardized mortality rates in regard to country NAP status, it was found that Central Asian countries exhibited the highest estimates. This suggests that, while Eastern Europe faces the highest overall burden, Central Asian countries may be particularly vulnerable in relation to the NAP and policy responses to AMR [13]. These findings highlight the complex nature of the AMR challenges across different parts of the former Soviet region. The disparity in AMR burden between high- and low-income countries highlights the importance of strengthening surveillance systems, improving laboratory infrastructure, and increasing capacity and training, particularly in resource-limited settings [14].

The post-Soviet space is one of the regions contributing to the AMR burden worldwide, mainly in terms of HIV and TB resistance [15,16,17]. Publications on the incidence of AMR and nosocomial infections are limited or not available in English. This barrier hinders the scientific community’s ability to discover, access, and utilize this information, leading to its unintentional neglect and underrepresentation in global studies [18]. Several studies have shed light on the AMR landscape in post-Soviet countries, highlighting concerning patterns. According to these studies, a retrospective analysis of infections from different wards in local hospitals showed that 70–80% of isolates had a multidrug resistance phenotype. In addition, multidrug-resistant species possessed a wide range of resistance genes and posed significant challenges for patient treatment and management in healthcare settings in these countries [19,20,21,22].

This systematic review assesses scholarly articles and publicly available information to characterize AMR surveillance and NAPs in former Soviet countries. These findings are needed to support evidence-based policymaking and facilitate improvements in existing AMR surveillance systems.

## 2. Results

### 2.1. Study Selection Description

The databases were used to identify 807 publications. A total of 334 duplicate papers were discovered and removed. The screening of titles and abstracts yielded 473 papers, of which 427 were excluded because they did not meet the inclusion criteria. The remaining 46 full-text studies were assessed for eligibility via critical appraisal checklists. The following 39 articles were excluded: studies of AMR prevalence; studies of the burden of AMR; studies that described mainly the infectious process and associated AMR; and studies that were not geographically relevant; studies that focused on the surveillance of sexually transmitted infections; and conference abstracts. A search for gray sources identified 23 publications, and the following qualitative assessments were included in the final list of included studies.

The final list of included studies comprised 7 peer-reviewed articles and 23 publications from gray sources. Among the seven peer-reviewed papers, five (71.4%) described the AMR surveillance system in the Russian Federation, one (14.3%) publication focused on the Georgian system, and another one (14.3%) described the AMR surveillance process in Belarus.

The gray source articles consisted of 11 (47.8%) country NAPs, 7 (30.4%) AMR-related legislative documents, and 3 (13%) country visit reports, all of which were published by credible organizations, institutions recognized for their expertise and reliability in providing health information, including the WHO, the Ministries of Health (MOH), the European Centre for Disease Prevention and Control (ECDC), and the National Public Health Agencies (NPHA). The last two (8.7%) results included Tracking Antimicrobial Resistance Country Self-Assessment Survey (TRACSS) responses for 2023 and the last joint report of EARS-Net and CAESAR 2023 (data for 2021). A summary of the included studies is given in Table S1.

### 2.2. National Action Plans

Among the 15 post-Soviet countries, 1 (6.7%) has no NAP or NAP under development—Estonia; 7 (46.7%) have developed NAPs—Armenia, Azerbaijan, Belarus, Latvia, Lithuania, Moldova, and Uzbekistan; and 6 (40%) have a NAP approved by the government that is being implemented—Georgia, Kazakhstan, Kyrgyzstan, Tajikistan, Turkmenistan, and Ukraine. In one country (6.7%), the implementation of the NAP is backed by financial support that has been integrated into the national plan and budget—Russia. Out of 14 developed NAPs, 11 (78.6%) are available on official sites (WHO, MOH, and NPHA) and are presented as a formal document with a clear title and an indicated year of adoption or timeline. Of the 11 publicly available NAPs, only 6 are up-to-date, i.e., will expire in 2024 or later.

The integration of the NAPs with the One Health approach was assessed as one of the elements of AMR surveillance. Among the 15 countries, 13 (86.7%) have established One Health coordination, although at various levels of integration and progress. In the remaining two countries (13.3%) (Armenia and Uzbekistan), there is no established coordination mechanism for AMR across different sectors.

Table 1 demonstrates the country’s NAP progress and its main features, as follows: One Health integration mechanism, development status, title and timeline, public availability, and general description of the AMR surveillance system.

### 2.3. National AMR Surveillance Systems

Out of 15 national AMR surveillance systems, 7 (46.7%) countries have national AMR surveillance at a standardized level, with AMR data collection and reference laboratory centers reporting sentinel sites—Belarus, Estonia, Kyrgyzstan, Latvia, Lithuania, Moldova, and Russia. In five (33.3%) countries, AMR data are collected locally, lacking coordination and effective quality management—Armenia, Azerbaijan, Kazakhstan, Turkmenistan, and Ukraine. Moreover, three (20%) countries collect AMR data nationally, which lack standardization or control—Georgia, Tajikistan, and Uzbekistan.

A national reference bacteriology laboratory (NRBL) has been established in 14 countries (93.3%), excluding Azerbaijan. The capacity to perform tests for priority bacteria is as follows: one (6.7%) none; six (40%) some bacteria; and eight (53.3%) countries responded that all listed bacteria can be identified and tested by the country’s NRBL. The capacity to test for priority fungi is as follows: two (13.3%) not reported; six (40%) none; three (20%) some fungi; and four (26.7%) countries responded that all fungi can be identified and tested for susceptibility in the country’s reference laboratory.

Standardized antibiotic susceptibility testing (AST) guidelines are compulsory and implemented in all laboratories in nine (60%) countries, whereas five (33.3%) indicated that only the NRBL and some sentinel sites adopted standardized AST, while one (6.7%) did not report on AST. External quality assessment (EQA) is implemented in all sites of 3 (20%) countries, whereas 11 (73.3%) have adopted EQA in some laboratories. None of the clinical bacteriology laboratories in Armenia implement EQA, according to the last TRACSS report [23].

**Figure 1:**
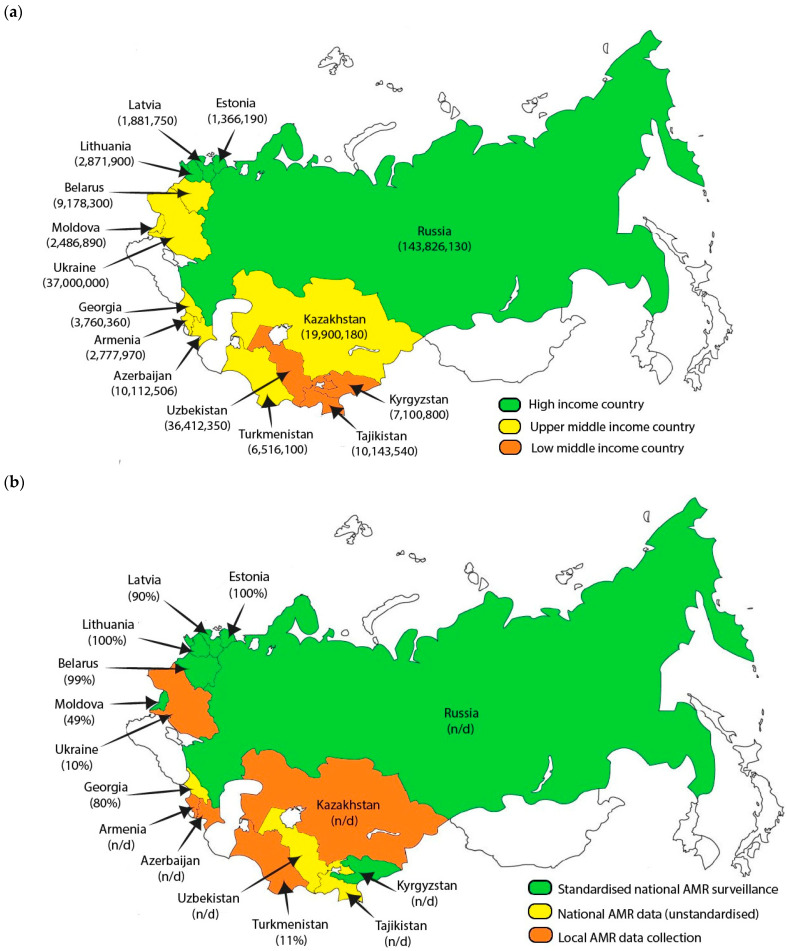
Map of countries under review. (**a**) The income level is categorized according to World Bank definitions as a high-income country (HIC), upper-middle-income country (UMIC), and lower-middle-income country (LMIC), with the population in parenthesis as of 2023 [12]; (**b**) Level of national AMR surveillance and estimated population coverage in parenthesis as of the latest CAESAR report [35]; n/d—no data available.

Figure 1 illustrates the countries under review. Figure 1a presents their respective income levels and population demographics; and Figure 1b displays the national AMR surveillance levels and estimated coverage of the population by national AMR surveillance.

Table 2 provides an overview of each country’s national AMR surveillance, covering the NRBL status, capacity for testing priority bacteria and fungi, use of standardized testing methods and adoption of EQA, and reporting to an international network, either EARS-Net or CAESAR.

### 2.4. International AMR Surveillance Systems

GLASS is a global surveillance network established by the WHO [36]. GLASS collaborates with regional networks, such as EARS-Net and CAESAR, which are Europe and Central Asia’s (CA) main regional AMR surveillance networks. The resistance data collected by EARS-Net and CAESAR are routinely incorporated into the GLASS database [35]. This integration provides a global picture of AMR trends and patterns.

Baltic countries are part of EARS-Net and have reported data on AMR since 2001. CAESAR includes European countries that are not part of EARS-Net and CA countries. The remaining 12 countries participate in CAESAR, although at different levels of involvement. CAESAR started collecting AMR data in 2014. The first two reports included AMR data for the preceding two years (e.g., the 2014 report included data for 2012 and 2013; and the 2016 report included data for 2014–2015). The subsequent reports included data collected from the previous year, with the 2017 report encompassing data from 2016, which continued for further years [35]. Therefore, annual CAESAR reports contain AMR data of member countries for the previous year or years.

In recent years, the number of former Soviet countries providing AMR data has gradually increased. In the first call, only Belarus submitted data in 2014 in the former Soviet region. In the following years, it increased from two (16.7%) to three (25%) between 2016 and 2017; in addition, during the next call, it increased from four (33.3%) in 2018 to five (41.7%) in 2019. Following that period, six (50%) countries reported in 2020. According to the latest CAESAR report (2023 year), 12 (100%) post-Soviet countries are engaged, and 8 (66.7%) submit AMR data to CAESAR [35]. The list of nonreporting countries includes Azerbaijan, Kyrgyzstan, Tajikistan, and Uzbekistan [35]. The full details of the countries’ participation and data submission in the CAESAR AMR surveillance network are given in Table S2.

AMRmap is a regional AMR surveillance network that primarily covers Russia. The network also includes some data from Belarus and Kazakhstan, particularly on epidemiologically significant genotypes [37]. However, AMRmap contains data from 1997, and the frequency and sustainability of data collection are not specified, indicating that it is an ongoing process [38].

### 2.5. Subgroup Analysis

Latvia and Lithuania have developed structured NAPs, whereas Estonia is developing one. All three aim to follow a multisectoral One Health approach addressing the human, animal, and environmental sectors [28,29,39]. Baltic countries actively participate in the European surveillance network and work toward improving their system, with Estonian overall AMR levels being below that of the European average [35]. All three countries report AMR data regularly, with an estimated population coverage of 90–100%. The geographical representativeness in all three countries is high, indicating adequate attention and devoted effort to AMR surveillance [35].

Moldova and Ukraine are not only geographically linked, as they share the Eastern European region, but are also committed to international and European standards [30,34]. Moldova’s NAP is more structured and strategic. While, Ukraine’s NAP has more action-oriented approach, with a strong focus on standardizing laboratory practices [34,40]. With respect to AMR surveillance coverage, Moldova covers half of the country with national surveillance and has high regional representativeness, while Ukraine has 10% population coverage and a medium level of regional representativeness [35].

Armenia, Azerbaijan, and Georgia form a unique group of Western Asian countries. Georgia’s NAP is more detailed and structured, having specific timelines and indicating financial backup [25]. According to the One Health report, there are limited regulations on AMR in Armenia [41]. Azerbaijan’s reported NAP is developed [23], but it is inaccessible. Azerbaijan also has not established an NRBL [23]. Georgia was one of the first countries in the former Soviet region to begin collecting and reporting AMR data and has high coverage. Whereas, Armenia only recently started reporting data to CAESAR [35].

Four CA countries (Kazakhstan, Kyrgyzstan, Tajikistan, and Turkmenistan) share common features in AMR-related legislation, such as strengthening national AMR surveillance and laboratory capacity, as well as the need to emphasize One Health collaboration. Owing to the unavailability of NAP, Uzbekistan’s initiatives on AMR legislation and surveillance functions were only assessed in the TRACSS report [23]. Kazakhstan’s AMR roadmap features as a set of measures for implementation [26]. The NAPs of Kyrgyzstan, Tajikistan, and Turkmenistan include several strategic points, as follows: AMR situation analysis, the structure of local surveillance, and defined objectives [27,32,33]. All CA countries have established NRBLs and have the capacity to test either all or some priority pathogens. National AMR surveillance population coverage is reported only for Turkmenistan (11%), whereas geographical representativeness is reported to be low for Kazakhstan and Turkmenistan [35].

Russia and Belarus have the most common characteristics, such as extensive national legislation and multisectoral approaches for addressing AMR [31,42,43]. Both countries’ policies emphasize AMR control and monitoring in healthcare facilities, particularly in risk-prone units, such as intensive care and surgery units [43,44]. The Russian NAP has a broader scope, covering a range of antimicrobial agents, including chemicals and disinfectants, as well as studying resistance mechanisms and finding alternative antimicrobials through research [31]. However, the Belarussian AMR policy has detailed clinical guidelines and administrative measures, such as restricting the use of reserve antibiotics without pharmacological approval [45]. NRBLs are established in each of the two countries. Russia has 12 reference centers affiliated with different research institutes, although they are unevenly distributed geographically [46]. The estimated population coverage for Belarus is 99%, but Russia’s coverage has not been revealed. The geographical representativeness of both countries was reported as high [35].

## 3. Discussion

The findings provide key insights into the post-Soviet region’s existing AMR surveillance and NAPs. Most countries have developed NAPs to address AMR, and all have established AMR surveillance systems across the region at various levels of advancement and scope. This includes functioning NRBLs and a coordinated network of sentinel laboratories performing standardized tests. This highlights the growing commitment to strengthen AMR surveillance systems among countries. However, the implementation of EQA is slightly behind, with only three countries having adopted EQA at all sites. Additionally, only two have integrated NAP and AMR surveillance approaches with One Health.

EQA is crucial for diagnostics. It helps to ensure that laboratories follow standardized procedures and interpret their results against reference metrics, which provides certainty that laboratories perform their functions well [47]. Traditional disk diffusion methods are widely used globally, especially in limited resource settings [48]. According to annual EQA reports, one-third of EARS-Net and one-half of CAESAR laboratories perform AST via disc diffusion testing methods [49,50]. While this method remains common, implementing a robust EQA program is essential to ensure the quality and reliability of AST results in the region.

The capacity to test for critical bacterial and fungal pathogens varies widely among the countries surveyed. For bacterial testing, eight countries have the capacity to test for all critical bacterial pathogens. However, testing for fungi lags behind. Only four countries in the region reported having the capacity to identify and provide susceptibility for all critical fungal pathogens. Additionally, of those that reported some fungi, the focus was exclusively of *Candida* species. The disparity among bacterial and fungal taxonomies highlights the unique challenges in fungal diagnostics, which primarily require molecular techniques for faster results and limited standardization [51,52].

Different surveillance networks adapt the WHO list of priority pathogens, resulting in variations in the bacteria reported. For instance, EARS-Net collected data of eight bacterial pathogens, whereas CAESAR collect nine, including *Salmonella* spp. [35]. The TRACSS survey includes 11 bacteria, which were considered critical because of their global importance [23]. Other factors influencing reporting practices include regional epidemiology, the diagnostic capabilities required for fastidious bacterial species, and the associated resource constraints that may affect an NRBL’s ability to test and report on all critical pathogens [53].

According to the review, there has been significant progress in NAP development across many regions worldwide [54], including Africa, where 35 out of 47 countries currently have NAPs on AMR [55]. However, implementation remains challenging for many countries, with gaps in establishing NRBLs and coordinating centers. This issue is especially demanding in developing countries and requires enhanced government engagement and leadership [14]. Iskandar et al. also argued that the “one size fits all” approach is not applicable. Therefore, an efficient action plan should start by understanding and recognizing each country’s context and features [14].

The level of One Health integration also varies widely worldwide. Delpy et al.’s scoping review revealed multisectoral integrated and domestically funded surveillance systems only in HICs [56]. One of the main shortcomings of One Health surveillance systems is a deficiency of harmonization between various elements, both within and across sectors. Another highlighted factor is that these integrated surveillance systems are rarely designed to assess the risks associated with AMR, while increasing awareness and developing policies [56].

A review by Gandra et al. analyzing AMR surveillance systems in eight high AMR burden low- and middle-income Asian countries revealed progress in establishing AMR surveillance programs in the last decade [57]. Alongside progress, the following common drawbacks were mentioned: poor laboratory facilities and lack of funding; low representativeness and population coverage; and biased AST results because of unstandardized testing and limited EQA adoption. This review also identified the difference in the implementation of AMR surveillance in the reviewed countries, e.g., Thailand, which has structured surveillance and was on the way to expanding its network, whereas, in Bangladesh and Laos, AMR surveillance was at the early levels of implementation [57].

Improved and reliable AMR surveillance data can provide the basis for evidence-based policy making, such as the implementation of stricter regulations on the sale of antimicrobial agents in both the human and veterinary sectors [58,59]. Continuous AMR surveillance allows us to assess the policy’s impact and, if necessary, to shift the regulations. Sharing AMR data is also emphasized to inform global challenges and strategies, as well as perspectives to guide the development of new antimicrobials and novel diagnostics [60].

This systematic review has several limitations. First, some of the data were taken from TRACSS responses, which were assessed and completed by country representatives. While the report provides insights, it does not fully capture the actual situation of AMR surveillance in these countries. The quality and completeness of TRACSS responses may vary and be inconsistent due to resource constraints or other associated issues. Moreover, data for each country may differ and be inconsistent between the latest CAESAR report (2023, data collected for 2021) and the TRACSS response (2023). The TRACSS data were utilized mainly during the review, being more recent. Another issue to consider is the limited clinical and epidemiological context. The available data do not expand beyond laboratory data and lack the clinical or demographic information vital to understanding AMR. There is a lack of published data covering the former Soviet region, especially peer-reviewed articles.

This review also has several strengths. To the best of our knowledge, following a thorough analysis of the literature on AMR surveillance in this area, this is the first systematic review covering the post-Soviet region. By examining the literature in English and local languages, we analyzed and summarized all available information on implementing NAPs and AMR surveillance activities in these countries.

## 4. Materials and Methods

### 4.1. Protocol and Registration

This systematic review was registered in the International Prospective Register of Systematic Reviews (PROSPERO) under the CRD42024537799 protocol.

### 4.2. Eligibility Criteria

The inclusion criteria included the following: publications on AMR surveillance published in peer-reviewed journals and gray literature addressing AMR in former Soviet countries (Armenia, Azerbaijan, Belarus, Estonia, Georgia, Kazakhstan, Kyrgyzstan, Latvia, Lithuania, Moldova, Russia, Tajikistan, Turkmenistan, Ukraine, and Uzbekistan). The exclusion criteria consisted of the following points: (i) surveillance activities related to animals, the environment, and food sources; (ii) publications addressing morphological or cellular analyses of bacterial resistance; (iii) papers addressing antimicrobial susceptibility patterns, trends, and aggregate resistance rates; (iv) publications that described tuberculosis, human immunodeficiency virus, and sexually transmitted infection surveillance systems; and (v) publications on surveillance outside of the post-Soviet region.

### 4.3. Search Strategies

PubMed, MEDLINE, Scopus, Embase, and CINAHL were utilized extensively to search for academic papers between 10 and 31 May 2024. The Nazarbayev University librarian was involved in developing a comprehensive search strategy to find all eligible studies. The search strategy is included in Table S3. The medical subject heading (MESH) terms were prepared via Boolean logical connectors on the basis of PubMed. Keywords were subsequently applied to other platforms, which were modified to be specific to each database. One reviewer (D.Z.) searched these databases using a search strategy. Search filters were applied from 1 January 2001 to the current day. The year 2001 was selected because of the publication of the WHO Strategy for Containment of AMR, the first document calling country members to action against AMR [61]. Additionally, we limited the scope to only studies that involved humans.

Considering the region’s particular characteristics, the authors also decided to search for articles in the Russian language. Therefore, the CyberLeninka database was considered a source of articles in the Russian language. Six carefully selected keywords with the same date range were used in the CyberLeninka search. All article titles that appeared were manually reviewed, and the full texts of the articles were retrieved for further analysis. The keywords for CyberLeninka are listed in Table S3.

Initially, two reviewers (D.Z. and Z.K.) performed a preliminary search for academic papers to evaluate the scope. This revealed the reality of the limited number of peer-reviewed articles on AMR surveillance in the region. As a result, it was also decided to consider reputable gray sources. The websites of each country’s official agencies and organizations were manually searched using the same keywords.

### 4.4. Study Selection

**Figure 2:**
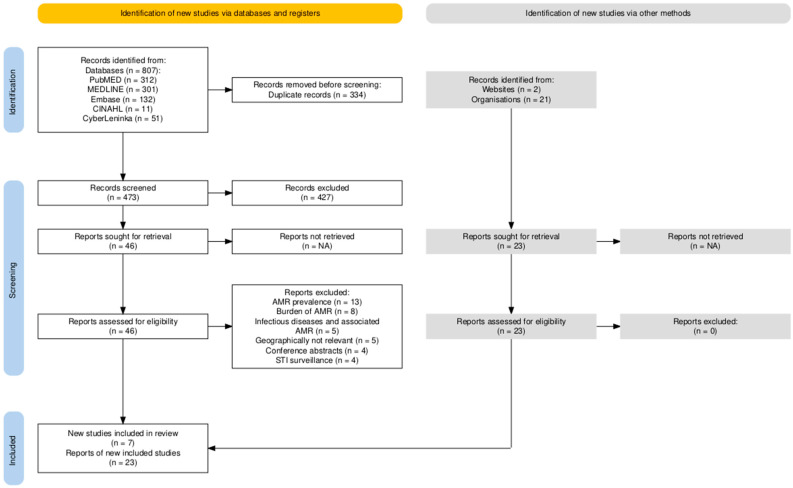
PRISMA 2020 flow diagram. NA—not applicable (all identified publications successfully retrieved).

At the beginning of the study, all of the publications, including scholarly and gray sources, were uploaded into a shared reference manager account in Zotero (version 6.0.36). After removing duplicates, two reviewers (D.Z. and Z.K.) started screening the titles and abstracts independently. Following screening, publications that did not meet the inclusion criteria were removed. Any disagreements on article eligibility were addressed, and a third reviewer (Y.S.) was consulted. The full texts of the eligible studies were subsequently retrieved for further assessment. The selection process followed the Preferred Reporting Items for Systematic Reviews and Meta-Analysis (PRISMA) 2020 checklist [62], illustrated in Figure 2.

### 4.5. Quality Assessment

A total of 46 peer-reviewed and 23 gray source publications were advanced to the following critical appraisal step. An Excel tabular format comprising all publications was developed to facilitate independent critical assessment and autonomous decisions on study quality.

The peer-reviewed articles were assessed via the Joanna Briggs Institute (JBI) appraisal checklist for qualitative studies, which comprises ten questions [63]. The AACODS checklist (authority, accuracy, coverage, objectivity, date, and significance) was used to assess the gray literature sources [64]. The AACODS checklist is composed of six blocks, each containing specific questions covering its respective areas. The reviewers answered “yes”, “no”, “unclear”, or “not applicable”. The articles that received two positive “yes” evaluations were marked as progressed and included in the review. If there were disagreements on the quality of the study and the inclusion decision, an independent reviewer (Y.S.) was involved to resolve the dispute.

### 4.6. Data Analysis

The information obtained from the included publications was retrieved and uploaded into Excel, summarized, and analyzed. First, legislative documents related to AMR were explored, as follows: (i) NAP existence, availability and timelines, and approaches for addressing AMR burdens and limitations; (ii) bylaws on the reference laboratories and relevant documents about the implementation of national AMR surveillance, methodology, coverage and representativeness, and frequency of reporting; and (iii) involvement in and reporting to international AMR surveillance networks.

To cover all NAPs and AMR bylaws of the post-Soviet space and make a comprehensive assessment, no language restrictions were applied. While official websites were searched, it was found that the policy documents of several countries were in the local language, which is not familiar to the authors of this paper. These were the NAPs of Armenia, Georgia, Latvia, Lithuania, Moldova, and Ukraine, which were translated with the help of an online translator. The NAPs of the remaining countries were available either in English or Russian.

The definitions for the categories in Table 1, which refer to the progress of each countries’ NAP, have been taken from relevant WHO documents [65,66], as follows: no NAP or NAP is under development—the country has not yet developed a NAP or is in the early stages of development; NAP developed—the country has drafted and completed a NAP document, but it has not yet been officially approved or implemented; NAP is approved by the government and is being implemented—the NAP document received government approval and implementation activities have begun; and NAP implementation funding is included in national plans and budgets—funding to implement the NAP has been incorporated into country’s official national planning and budgeting, indicating government commitment to NAP implementation.

The countrie’s multisectoral and One Health approaches were categorized into two variables, considering the unclear definitions of TRACSS responses, as follows: no formal One Health coordination—a lack of formal multisectoral coordination; and established One Health coordination—various levels of One Health approach, including multisectoral coordination mechanism on AMR with government leadership, with technical groups working and collaborating on common goals and integrated approaches.

Population coverage percentage was estimated by national AMR focal points according to the population covered by the hospitals included in national surveillance. The geographical representativeness indicates how the surveillance system reflects the different areas of the country. Both were provided by the countries to the respective network [35].

We divided all countries into subgroups, taking into account the geographical and structural frameworks, as follows: Baltic countries (Estonia, Latvia, and Lithuania), Western Asia (Armenia, Azerbaijan, and Georgia), Central Asia (Kazakhstan, Kyrgyzstan, Tajikistan, Turkmenistan, and Uzbekistan), Eastern Europe (Moldova and Ukraine), Russia, and Belarus. A comparative and descriptive analysis of AMR governance and surveillance among these countries and subgroups was conducted. The assessment of NAPs was performed according to the WHO GAP [7], whereas the evaluation of national AMR surveillance was performed according to the CAESAR manual [9].

## 5. Conclusions

This systematic review provides insights into the progress achieved by post-Soviet countries regarding national AMR surveillance. According to all of the sources, TRACSS, and CAESAR reports, the countries have significant disparity, with the three Baltic countries being the most advanced. Russia, Belarus, Moldova, and Georgia have more sophisticated AMR surveillance systems, while the remaining countries display room for improvement. Azerbaijan needs to establish an NRBL and develop the capacity to perform testing, which would be beneficial for CA countries to start reporting AMR data, considering their underrepresentation in most CAESAR reports. Most of the countries (CA, Russia, Caucasus, and Ukraine) still need to improve coverage and regional representation of AMR data and increase the adoption of EQA standards.

All countries in the region have demonstrated their commitment to strengthening AMR surveillance by joining international networks. However, much effort is still needed to further progress and align national AMR surveillance with global best practices.

## Figures and Tables

**Table 1:** Progress of the NAP and AMR surveillance in the post-Soviet region.

Country	Multisector and One Health Collaboration/Coordination [23]	Progress with NAP Development [23]	Title of the NAP	Year of Adoption, Timeline	Availability of the NAP on Official Websites	National AMR Surveillance System [23]
Armenia	No formal One Health coordination	NAP developed	National Action Plan on Antimicrobial Resistance [24]	2015–2020	available	Local AMR data collection
Azerbaijan	Established One Health coordination	NAP developed	n/d	n/d	Not available	Local AMR data collection
Belarus	Established One Health coordination	NAP developed	n/d	n/d	Not available	Standardized national AMR surveillance
Estonia	Established One Health coordination	No NAP or NAP under development	n/d	n/d	Not available	Standardized national AMR surveillance
Georgia	Established One Health coordination	NAP is approved by the government and is being implemented	National Strategy Against Antimicrobial Resistance [25]	2017–2020	Available	National AMR data (unstandardized)
Kazakhstan	Established One Health coordination	NAP is approved by the government and is being implemented	Roadmap “On Measures to Contain Antimicrobial Resistance in the Republic of Kazakhstan for 2023–2027” [26]	2023–2027	Available	Local AMR data collection
Kyrgyzstan	Established One Health coordination	NAP is approved by the government and is being implemented	Containment of Antimicrobial Resistance in the Kyrgyz Republic for 2022–2025 [27]	2022–2025	Available	Standardized national AMR surveillance
Latvia	Established One Health coordination	NAP developed	“One Health” Plan for Containing Antimicrobial Resistance and Prudent Use of Antibiotics for 2019–2020 [28]	2019–2020	Available	Standardized national AMR surveillance
Lithuania	Established One Health coordination	NAP developed	Order on the prevention and control of the spread of antimicrobial-resistant microorganisms and hospital infections [29]	2023–2027	Available	Standardized national AMR surveillance
Republic of Moldova	Established One Health coordination	NAP developed	Order on the approval of the National Program for surveillance and control of antimicrobial resistance for the years 2023–2027 [30]	2023–2027	Available	Standardized national AMR surveillance
Russian Federation	Established One Health coordination	NAP implementation funding is included in national plans and budgets	Action Plan for the Implementation of the Strategy for the Prevention of the Spread of Antimicrobial Resistance in the Russian Federation by 2030 [31]	2019–2024	Available	Standardized national AMR surveillance
Tajikistan	Established One Health coordination	NAP is approved by the government and is being implemented	National Action Plan to Tackle Antimicrobial Resistance in the Republic of Tajikistan [32]	2018	Available	National AMR data (unstandardized)
Turkmenistan	Established One Health coordination	NAP is approved by the government and is being implemented	National Strategy for Containment of Antimicrobial Resistance in Turkmenistan [33]	2017–2025	Available	Local AMR data collection
Ukraine	Established One Health coordination	NAP is approved by the government and is being implemented	National action plan on combating the resistance to antimicrobial drugs [34]	2019	Available	Local AMR data collection
Uzbekistan	No formal One Health coordination	NAP developed	n/d	n/d	Not available	National AMR data (unstandardized)

NAP—national action plan; AMR—antimicrobial resistance; n/d—no data available.

**Table 2:** Description of national AMR surveillance systems.

Country	NRBL Status [23]	NRBL Performing AST Testing of Critically Important Bacteria * [23]	NRL Performing Identification and AST Testing of Critically Important Fungi ** [23]	Use of Standardized AST Guidelines in NRBL and Local Labs [23]	EQA Program is Compulsory and/or Implemented in NRBL and Local Labs [23]	Reporting to International Network [35]
Armenia	established	◊	✕	✓	✕	✓
Azerbaijan	not established	✕	n/d	n/d	◊	✕
Belarus	established	✓	n/d	✓	◊	✓
Estonia	established	✓	✕	✓	✓	✓
Georgia	established	✓	✕	◊	◊	✓
Kazakhstan	established	◊	✕	✓	◊	✓
Kyrgyzstan	established	◊	◊	✓	◊	✕
Latvia	established	✓	✓	✓	◊	✓
Lithuania	established	✓	✕	✓	✓	✓
Republic of Moldova	established	◊	✕	◊	◊	✓
Russian Federation	established	✓	✓	✓	✓	✓
Tajikistan	established	✓	✓	◊	◊	✕
Turkmenistan	established	◊	◊	✓	◊	✓
Ukraine	established	◊	◊	◊	◊	✓
Uzbekistan	established	✓	✓	◊	◊	✕

✓—all/yes; ◊—some; ✕—none/no; * List of 11 critically important bacteria (*Acinetobacter baumannii*, *Pseudomonas aeruginosa*, *Enterobacteriales: E. coli*, *Klebsiella*, *Proteus*; *Enterococcus faecium*, *Staphylococcus aureus*, *Campylobacter* spp., *Salmonellae* spp., *Neisseria gonorrhoeae*, *Streptococcus pneumoniae*, *Haemophilus influenzae*, and *Shigella* spp.); ** List of critically important fungi (Candida spp. and Aspergillus spp.); n/d—unknown/no data available (some countries responded as unknown or left the question unanswered); NRBL—national reference bacteriology laboratory; NRL—national reference laboratory; AST—antibiotic susceptibility testing; EQA—external quality assessment.

## Data Availability

All data sources and links are provided in the Supplementary Materials.

## Supplementary Materials

The following supporting information can be downloaded at: https://www.mdpi.com/article/10.3390/antibiotics13121129/s1, Table S1: Included studies; Table S2: Engagement status and reporting of countries in the CAESAR network; Table S3: Search strategy.
