# Supplementary material for: Antimicrobial Resistance Surveillance in Post-Soviet Countries: A Systematic Review
Source: Antibiotics (Basel). 2024 Nov 25;13(12):1129. doi: 10.3390/antibiotics13121129 (PMC11672431; doi:10.3390/antibiotics13121129)
Supplement: Supplementary file 1 [file antibiotics-13-01129-s001.zip › Supplementary materials_Table_S1.pdf]

Supplementary file

Table S1. Included studies

№	Authors, date	Title	Publication design	Main objective	Setting	Link
	**Peer-reviewed articles**					
1	Malania et al., 2021	Setting up laboratory-based antimicrobial resistance surveillance in low- and middle-income countries: Lessons learned from Georgia. Clinical Microbiology and Infection: The Official Publication of the European Society of Clinical Microbiology and Infectious Diseases.	Review	To provide an overview of AMR surveillance components in LMICs and a description of surveillance activities in Georgia.	Georgia	https://doi.org/10.1016/j.cmi.2021.05.027
2	Titov et al., 2013	Monitoring of antimicrobial resistance as the element of the surveillance system and the key direction of the European strategic action plan upon the problem.	Review	To narrate AMR surveillance system in the country, its legislation, and actions taken to improve surveillance.	Belarus	https://belriem.by/informatsiya/naseleniyu/problema-lekarstvennoy-ustoychivosti/
3	Orechov et al., 2023	Antimicrobial Resistance: A Risk Factor for the Biosafety System.	Review	To narrate AMR surveillance legislation and framework in the country.	Russian Federation	https://doi.org/10.30895/2312-7821-2023-11-3-336-347
4	Petrova et al., 2022	Experience in implementing the AMRcloud online platform for local antimicrobial resistance surveillance in a tertiary care hospital.	Review and implementation study	To create a platform for AMR monitoring and implement it in local hospitals. It also describe Interagency Programs the features, strengths, and limitations of online platforms for monitoring AMR.	Russian Federation	https://doi.org/10.36488/cmac.2022.1.39-46
5	Kuzmenkov et al., 2021	AMRmap–antibiotic resistance surveillance system in Russia.	Review	An overview of the principles and features of the AMRmap platform.	Russian Federation	DOI:10.35488/cmac.2021.2.198-204
6	Svetlichnaya at al., 2014	Microbiological Monitoring in Epidemiological Surveillance for Hospital Infections.	Cross-sectional study	To describe local AMR surveillance efforts in St Petersburg hospitals and at the regional level.	Russian Federation	https://cyberleninka.ru/article/n/mikrobiologicheskiy-monitoring-v-sisteme-epidemiologicheskogo-nadzora-za-gospitalnymi-infektsiyami
7	Kuzmenkov et al., 2020	Antimicrobial resistance monitoring: A review of information resources.	Review	To outline the strengths and limitations of international and locally implemented AMR surveillance networks.	Russian Federation	DOI:10.20538/1682-0363-2020-2-163-170

	**Gray sources**					
8	Breen et al., 2021	An assessment of One Health operations and capacities in Armenia.	Country visit report	To describe the country's progress on biosurveillance and biodefense activities through the One Health approach.	Armenia	https://www.ecohealthalliance.org/wp-content/uploads/2023/11/One-Health-Armenia-Report_FINAL_english.pdf
9	MOH Armenia, 2015	National Action Plan on Antimicrobial Resistance.	National legislation	To address AMR by introducing policy and planned activities.	Armenia	https://www.who.int/publications/m/item/armenia-national-action-plan-to-combat-antimicrobial-resistance
10	MOH Belarus, 2003	Order on the establishment of a reference center for monitoring resistance to antibiotics, antiseptics, and disinfectants of clinically important microorganisms.	Order	To approve and assign the national reference laboratory and its functions.	Belarus	https://belriem.by/informatsiya/naseleniyu/problema-lekarstvennoy-ustoychivosti/
11	MOH Belarus, 2012	Order on the approval of the instruction on the procedure for monitoring of resistance of clinically relevant microorganisms to antibacterial drugs in healthcare organizations.	Order	Guidelines on the organization of AMR surveillance in healthcare facilities.	Belarus	https://belriem.by/informatsiya/naseleniyu/problema-lekarstvennoy-ustoychivosti/
12	MOH Belarus, 2015	Order on measures to reduce antimicrobial resistance in microorganisms.	Order	List of actions to reduce AMR, with activities targeting infection prevention and strengthening AMR knowledge and surveillance networks.	Belarus	https://belriem.by/informatsiya/naseleniyu/problema-lekarstvennoy-ustoychivosti/
13	ECDC, 2019	Joint report in respect of a One Health country visit to Estonia to discuss policies relating to antimicrobial resistance.	Country visit report	To support Estonia in developing and implementing policies and legislation to tackle AMR.	Estonia	https://www.ecdc.europa.eu/en/publications-data/ecdc-and-european-commission-country-visit-estonia-discuss-policies-relating
14	MOH Georgia, 2017	National Strategy Against Antimicrobial Resistance	National strategy	To address AMR by introducing policy and planned activities.	Georgia	https://www.fao.org/faolex/results/details/en/c/LEX-FAOC196391/
15	MOH Kazakhstan, 2022	Roadmap "On Measures to Contain Antimicrobial Resistance in the Republic of Kazakhstan for 2023-2027"	Order on measures to contain AMR	To address AMR by introducing policy and planned activities.	Kazakhstan	https://hls.kz/ru/piik-ru

16	MOH and MOA Kyrgyzstan, 2022	Interagency Program "Containment of Antimicrobial Resistance in the Kyrgyz Republic for 2022-2025."	National legislation	To address AMR by introducing policy and planned activities.	Kyrgyzstan	The National Action Plan on AMR for 2022-2025 of the Kyrgyz Republic. | FAOLEX
17	ECDC, 2011	Country mission Latvia: antimicrobial resistance.	Country visit report	To discuss and evaluate the country's activities for the control of AMR.	Latvia	https://www.ecdc.europa.eu/en/publications-data/ecdc-country-visit-latvia-discuss-antimicrobial-resistance-issues
18	MOH Latvia, 2019	"One Health" Plan for Containing Antimicrobial Resistance and Prudent Use of Antibiotics for 2019-2020	National action plan	To address AMR by introducing policy and planned activities.	Latvia	https://www.fao.org/faolex/results/details/en/c/LEX-FAOC196392/
19	MOH Lithuania, 2023	Order on the prevention and control of the spread of antimicrobial-resistant microorganisms and hospital infections	Order	To address AMR by introducing policy and planned activities	Lithuania	https://www.e-tar.lt/portal/lt/legalAct/200be3d0a7b611ed8df094f359a60216
20	MOH Lithuania, 2013	Order on the surveillance of resistance of clinically and epidemiologically important microorganisms to antimicrobial agents and description of the procedure for the collection, accumulation, analysis, and reporting of data on the resistance of microorganisms to antimicrobial agents.	Order	To describe the procedures for collecting, storing, and providing information on antimicrobial resistance of clinically and epidemiologically relevant microorganisms.	Lithuania	https://www.e-tar.lt/portal/lt/legalAct/c9324b40723011e3bd0ecaffd80c672a
21	MOH Moldova, 2023	Order on the approval of the National Program for surveillance and control of antimicrobial resistance for the years 2023-2027.	Order	To address AMR by introducing policy and planned activities.	Moldova	https://gov.md/ro/content/hg-proiect-de-hotarare-cu-privire-la-aprobarea-programului-national-pentru-supravegherea-si
22	MOH Russia, 2017	Strategy for the Prevention of the Spread of Antimicrobial Resistance in the Russian Federation by 2030.	Governmental Strategy	To prevent and limit the spread of microbial resistance to antimicrobial agents, plant pests, and antimicrobial chemical and biological agents, including pesticides.	Russia	http://government.ru/docs/29477/

23	MOH Russia, 2019	Action Plan for the Implementation of the Strategy for the Prevention of the Spread of Antimicrobial Resistance in the Russian Federation by 2030.	National action plan	To address AMR and implement the measures proposed in the strategy.	Russia	http://government.ru/docs/36320/
24	MOH Russia, 2020	Order on approval of the list of reference centers for specific types of medical activities in order to ensure systematic monitoring of the spread of antimicrobial resistance.	Order	To approve and assign the list of reference centers of the laboratory.	Russia	https://mycology.szgmu.ru/files/20201224_1366_%D0%9F%D1%80%D0%B8%D0%BA%D0%B0%D0%B7_%D0%A0%D0%B5%D1%84%D0%B5%D1%80%D0%B5%D0%BD%D1%81-%D1%86%D0%B5%D0%BD%D1%82%D1%80%D1%8B.pdf
25	MOH, AgroMinistry and Ministry of food security Tajikistan, 2018	National Action Plan to Tackle Antimicrobial Resistance in the Republic of Tajikistan.	National action plan	To address AMR by introducing policy and planned activities.	Tajikistan	https://www.who.int/publications/m/item/tajikistan-national-action-plan-to-tackle-antimicrobial-resistance-in-the-republic-of-tajikistan
26	Ministry of Health and medical industry Turkmenistan, 2017	National Strategy for Containment of Antimicrobial Resistance in Turkmenistan.	National Strategy	To address AMR by introducing policy and planned activities.	Turkmenistan	https://www.who.int/publications/m/item/turkmenistan-national-strategy-for-containment-of-antimicrobial-resistance-in-turkmenistan
27	MOH Ukraine, 2019	National Action Plan on Combating the Antimicrobial Resistance to Antimicrobial Drugs.	National action plan	To address AMR by introducing policy and planned activities.	Ukraine	https://www.kmu.gov.ua/npas/proogo-planu-dij-shchodo-borotbi-iz-stijkistyu-do-protimikrobnih-preparativ
28	MOH Ukraine, 2007	On the approval of methodical instructions "Determining the sensitivity of microorganisms to antibacterial drugs."	National legislation	To approve methodical instructions for AST testing.	Ukraine	https://zakon.rada.gov.ua/rada/show/v0167282-07?lang=en#Text
29	WHO, UNEP, FAO, and WOAH, 2023	Tracking antimicrobial resistance country self-assessment survey.	Self-assessment survey results	To monitor the progress made by countries in tackling AMR (NAPs and AMR surveillance system implementation).	All countries	https://amrcountryprogress.org/#/map-view

30	ECDC and WHO, 2023	Antimicrobial resistance surveillance in Europe 2023, CAESAR+EARS-Net report (2021 data).	Report	To present AMR from invasive infections in Europe.	All countries	https://www.ecdc.europa.eu/en/publications-data/antimicrobial-resistance-surveillance-europe-2023-2021-data
