# Supplementary material for: Antimicrobial Resistance Surveillance in Post-Soviet Countries: A Systematic Review
Source: Antibiotics (Basel). 2024 Nov 25;13(12):1129. doi: 10.3390/antibiotics13121129 (PMC11672431; doi:10.3390/antibiotics13121129)
Supplement: Supplementary file 1 [file antibiotics-13-01129-s001.zip › Supplementary materials_Table_S2.pdf]

Supplementary file

Table S2. Engagement and reporting status of countries in the CAESAR network

**Countries**	**CAESAR 2014 (data for 2012–2013)**		**CAESAR 2016 (data for 2014–2015)**		**CAESAR 2017 (data for 2016)**		**CAESAR 2018 (data for 2017)**		**CAESAR 2019 (data for 2018)**		**CAESAR 2020 (data for 2019)**		**CAESAR + EARS-Net 2022 (data for 2020)**		**CAESAR + EARS-Net 2023 (data for 2021)**	
	engaged	reported	engaged	reported	engaged	reported	engaged	reported	engaged	reported	engaged	reported	engaged	reported	engaged	reported
**Armenia**	+	-	+	-	+	-	+	-	+	+	+	+	+	-	+	+
**Azerbaijan**	+	-	+	-	+	-	+	-	+	-	+	-	+	-	+	-
**Belarus**	+	+	+	+	+	+	+	+	+	+	+	+	+	+	+	+
**Georgia**	+	-	+	-	+	+	+	+	+	+	+	+	+	+	+	+
**Kazakhstan**	-	-	+	-	+	-	+	-	+	-	+	-	+	-	+	+
**Kyrgyzstan**	+	-	+	-	+	-	+	-	+	-	+	-	+	-	+	-
**Moldova**	+	-	+	-	+	-	+	-	+	-	+	+	+	+	+	+
**Russia**	+	-	+	+	+	+	+	+	+	+	+	+	+	+	+	+
**Tajikistan**	+	-	+	-	+	-	+	-	+	-	+	-	+	-	+	-
**Turkmenistan**	+	-	+	-	+	-	+	-	+	-	+	-	+	-	+	+
**Ukraine**	+	-	+	-	+	-	+	+	+	+	+	+	+	+	+	+
**Uzbekistan**	+	-	+	-	+	-	+	-	+	-	+	-	+	-	+	-
**overall**	11	1	12	2	12	3	12	4	12	5	12	6	12	5	12	8

"+"–indicates engagement/submission of AMR data.

"-"–indicates lack of engagement/submission of AMR data.
