# Supplementary material for: Antimicrobial Resistance Surveillance in Post-Soviet Countries: A Systematic Review
Source: Antibiotics (Basel). 2024 Nov 25;13(12):1129. doi: 10.3390/antibiotics13121129 (PMC11672431; doi:10.3390/antibiotics13121129)
Supplement: Supplementary file 1 [file antibiotics-13-01129-s001.zip › Supplementary materials_Table_S3.pdf]

Supplementary file

Table S3. Search strategy

**PICO framework**	**Population or Problem**–Antimicrobial resistance<br>**Intervention**–Surveillance<br>**Comparator**–Not applicable<br>**Outcome**–Surveillance system design, applied methodology, characteristics, and coverage of the surveillance system**.**
**Concept 1** Antimicrobial resistance	(("Drug Resistance, Microbial"[Mesh]) OR (“Drug Resistance"[Mesh] OR "Drug Resistance, Multiple, Bacterial"[Mesh] OR "Drug Resistance, Bacterial"[Mesh] OR "Drug Resistance, Multiple"[Mesh] OR “Drug Resistance, Microbial"[Mesh])) OR "Microbial Sensitivity Tests"[Mesh] OR **“**Antimicrobial resistance*” OR “drug resistance*” OR “antibiotic resistance*” OR “bacterial resistance*”
**Concept 2** Surveillance	"Public Health Surveillance"[Mesh] OR "Sentinel Surveillance"[Mesh] OR "Population Surveillance"[Mesh] OR "Epidemiological Monitoring"[Mesh] OR “Antimicrobial resistance surveillance” OR “resistance monitoring” OR “sentinel surveillance” OR “epidemiological monitoring” OR “framework*” OR “action plan*”
**Concept 3** Post-Soviet countries	((("USSR"[Mesh] OR "Dagestan"[Mesh] OR "Armenia"[Mesh]) OR "Baltic States"[Mesh]) OR (“Asia, Central"[Mesh] OR "Siberia"[Mesh] OR “Georgia (Republic)"[Mesh])) OR "Azerbaijan"[Mesh] OR “Soviet Union” OR “Post-Soviet countr*” OR “Baltic countr*” OR “Central Asia” OR Caucasus OR Estonia OR Latvia OR Lithuania OR Kazakhstan OR Kyrgyzstan OR Russia OR Belarus OR Moldova OR Ukraine OR Georgia OR Tajikistan OR Turkmenistan OR Armenia OR Georgia OR Azerbaijan
**CyberLeninka**	"антимикробная резистентность" (antimicrobial resistance)<br>"надзор" (surveillance)<br>"эпидемиологический надзор" (epidemiological surveillance)<br>"мониторинг" (monitoring)<br>"устойчивость к противомикробным препаратам" (resistance to antimicrobial drugs)<br>"устойчивость к антибиотикам" (resistance to antibiotics)
